# Endometrial Preparation for Women Undergoing Embryo Transfer Frozen-Thawed Embryo Transfer With and Without Pretreatment With Gonadotropin Releasing Hormone Agonists 

**Published:** 2018-12

**Authors:** Shohreh Movahedi, Ashraf Aleyasin, Marzieh Agahosseini, Leili Safdarian, Sahar Abroshan, Sepideh Khodaverdi, Parvin Fallahi

**Affiliations:** 1 Department of Infertility of Shariati Hospital, Tehran University of Medical Sciences, Tehran, Iran; 2 Department of Obstetrics and Gynecology, Tehran University of Medical Sciences, Tehran, Iran; 3 Department of Obstetrics and Gynecology, Fellowship in Minimally Invasive Gynecology Surgery (FMIGS),Endometriosis Research Center of Rasoul Akram Hospital, Iran University of Medical Sciences , Tehran, Iran; 4 Department of Infertility, Shariati Hospital, Tehran University of Medical Sciences, Tehran, Iran

**Keywords:** Endometrial Preparation, Embryo Transfer, Frozen-Thawed, Gonadotropin, Hormone Agonists, Women

## Abstract

**Objective:** To evaluate the efficacy of endometrial preparation by exogenous steroids, with and without pretreatment by the use of GnRH agonist.

**Materials and methods:** This randomized interventional study was conducted on 100 women who underwent a FTET that were randomly assigned to receive GnRH agonist (buserelin) in the luteal phase or no receive this medication. In both groups endometrial preparation was achieved by the use of estradiol valerate pill started from the second day of the menstruation and used every day, with an initial dose of 2mg/d and every 3 days increased to 4 mg/d and 6 mg/d, respectively. Endometrial thickness was evaluated by vaginal ultrasound. Forty eight hours after beginning of progesterone administration 2 to 3 embryos surviving in freezing procedure were transferred.

**Results:** the two groups were similar in mean age, body mass index, duration of infertility, type of infertility, number of embryos transferred and endometrial thickness on the day of beginning progesterone therapy. Comparing outcome of FTET between the two groups scheduled for receiving GnRH agonist showed no significant difference in the rate of implantation (6.7% versus 10.0%), the rate of chemical pregnancy (21.7% versus 22.5%), clinical pregnancy rate (15.0% versus 17.5%), and also ongoing pregnancy (13.3% versus 12.5%).

**Conclusion:** Endometrial preparation for FTET using GnRH agonists appears to be as effective as FTET without administrating these agonists.

## Introduction

Frozen-thawed embryo transfer (FTET) is an applicable procedure to storing and transferring excess embryos achieved within in vitro fertilization (IVF) (1). By developing recent laboratory techniques, the possibility has been provided for increase in FT embryo transfer cycles. One of the best practices for prevention of multiple pregnancies in IVF cycles includes transferring a single embryo followed by freezing all surplus embryos (2). In this regard, the final goal of FTET is endometrial preparation to increase the chance for pregnancy in a single stimulated cycle. In fact, the main advantage of using FTET is to increase the cumulative pregnancy rate in addition to minimize the cost and to shorten the time duration of the procedure leading successful pregnancy (3). Therefore, the aim of studies should be focused on increasing the success rate of FTET cycles by appropriately preparing the endometrium in these cycles. Recently, artificially preparing the endometrium through administration of gonadotropin-releasing hormone agonist has been taken into consideration (4). In fact, hormonal preparation of the endometrium is now a good alternative for achieving proper outcome in infertile women who candidate for FTET procedure (5). Multiple drugs and roots of administration have been introduced in order to optimize the success rate of FTET. Recently, the use of gonadotropin releasing hormone (GnRH) agonist usually in a depot form is now considered as the best practice for suppression of ovarian function to avoid interposition with stimulation of exogenous estrogenic stimulation and proliferation of the endometrium (6). Currently, most of the protocols involve pituitary down-regulation with a GnRH agonist to avoid spontaneous ovulation before sequential administration of 17-B estradiol and progesterone (7). The programmed cycle using GnRH agonist followed by estrogens and progesterone has shown similar pregnancy rates in thawed embryos and replacement during the natural cycle (8). The disadvantages of pretreatment with GnRH agonist include the high cost of the GnRH analogue, the risk of hypo-estrogenic side effects before hormonal replacement and long preparation. Frozen-thawed embryo transfer (FET) has been successfully performed during a natural cycle after spontaneous ovulation (7-9) or after artificial preparation of endometrium with exogenous steroids (7-9). The present study aimed to evaluate the efficacy of endometrial preparation by exogenous steroids, with and without pretreatment by the use of GnRH agonist.

## Materials and methods

The study protocol was approved by the Ethics committees of the university (referred to the corresponding author's affiliation; IRCT Registration ID: IRCT201109224572N2) and the study have been performed in accordance with the ethical standards as laid down in the 1964 Declaration of Helsinki and its later amendments or comparable ethical standards. From July2008 to April 2009, 100 women with the functioning ovary and with a normal cavity of uterus were enrolled in the study. All participants were 25 to 38 years old which were eligible for infertility treatments. The analysis was confined to FET cycles which were not donor. All patients who had previously undergone intracytoplasmic sperm injection (ICSI) cycle with embryo cryopreservation and the transfer of their frozen-thawed embryos prospectively participated in this interventional study. We excluded patients above 39 years old, and the ones whose FSH was above 11, had endometriosis and hypothalamic amenorrhea. 

After obtaining informed consent, the patients were randomly allocated into two treatment groups.In study group (A) 60 patients used oral contraceptive-LD (manufactured by Aburaihan Co., Tehran- Iran) the month before the embryo transfer, GnRH agonist suppression (buserelin: S uperfact-manufactured by Aventis Pharma Deutschland) 0.5mg/day was administered from the day 21 of the cycle. The control group (B) was composed of 40 patients who did not use GnRH agonist. In both groups endometrial preparation was achieved by the use of estradiol valerate pill2mg (manufactured by Aburaihan Co. Tehran-Iran), which were started from the second day of the menstruation and were used every day, with initial dose of 2mg/d and after 3 days increased to 4 mg/d and after 3 days again increased to 6 mg/d. Trans-vaginal ultrasound (TVU) was performed on the 13^th ^day of estradiol treatment. Stimulation characteristics and protocol for the collection ICSI cycle were standard. After oocyte retrieval, handling of the gametes, including performance ICSI, was per routine protocols (10). Embryos obtained by ICSI procedures were cryopreserved. Cryopreservation was done by using routine examination of: 1) the number of blastomeres; 2) the degree of fragmentation; and 3) the uniformity of blastomeres.

Embryo morphology was scored as: grade A, ≤ 10% fragmentation and equal blastomeres; grade B, 10% to ≤ 30% fragmentation and equal blastomeres; grade C: 30%–50% fragmentation and/or unequal blastomeres; grade D: ≥ 50% fragmentation. Our strategy was to freeze the supernumerary embryos only if they exhibited a favorable grading with ≤ 30% fragmentation and regular blastomeres, since the cryosurvival rate is related to the initial quality of the embryo (11). At the time of vitrification embryos were transferred into equilibration solution consisting of 7.5% (v/v) ethylene glycol (EG) and 7.5% (v/v) DMSO dissolved in TCM199 supplemented with 20% synthetic serum substitute (SSS) at 27°C for 5-15 minutes. 

After an initial shrinkage, embryos regained their original volume, and were transferred into three 20 μl drops of vitrification solution consisting of 15% (v/v) EG and 15%(v/v) DMSO and 0.5 mol/l sucrose dissolved in TCM199 supplemented with 20% SSS, After incubation for 20 seconds in each drop respectively, embryos were loaded into CryoTips or on Cryotops and plunged into liquid nitrogen.

On the day of FET, the embryos were rapidly thawed and sequentially rehydrated at room temperature in 1.0 M PROH/0.2 M sucrose (5 minutes), 0.5 M PROH/0.2 M sucrose (5 minutes), 0.2 M sucrose (10 minutes), and ET freezing and thawing media (5 minutes). Thawed embryos were transferred to 37°C and evaluated under × 200 magnifications for blastomere survival. Embryo grading was performed according to predetermined criteria (10). 

If endometrial thickness (ET) was measured (EM) ≥ 7mm, progesterone was added to the estradiol regimen and if the ET was < 7 mm,2mgestradiol valerate was added for four days before repeating TVU and starting progesterone (cyclogest 400 mg pessaries manufactured by Actavis, Barnstaple, UK) was used vaginally, at a dose of 800 mg/d.

After thawing, embryos surviving the freezing procedure with ≤ 30% fragmentation and regular blastomeres and2 to 3 embryos were transferred via trans-cervical route with Wallace catheter (SIMS Portex Ltd., Hythe, Kent, UK), 48 hours after the beginning of progesterone administration.

Hormonal treatment was continued at least until the performance of pregnancy test 15 days after the transfer. By occurrence of pregnancy, hormonal treatment was continued at least for 8 weeks in this period. The cycle outcomes were categorized as pregnancy (positive serum β-hCG on day 15 after ET) and clinical pregnancy (positive fetal heart on trans-vaginal ultrasound). The implantation rate was calculated as the number of gestational sacs identified on trans-vaginal ultrasound per number of frozen embryos transferred.

Results were presented as mean ± standard deviation (SD) for quantitative variables and were summarized by absolute frequencies and percentages for categorical variables. Normality of data was analyzed using the Kolmogorov-Smirnoff test. Categorical variables were compared using chi-square test or Fisher's exact test when more than 20% of cells with expected count of less than 5 were observed. Quantitative variables were also compared with t test or Mann- Whitney U test. To determine main determinants of disease prognosis, the multivariate regression modeling was employed. For the statistical analysis, the statistical software SPSS version 16.0 for windows (SPSS Inc., Chicago, IL) was used. P values of 0.05 or less were considered statistically significant.

## Results

As shown in table 1 with regard to compare baseline characteristics of the groups with and without administrating GnRH agonist, the two groups were similar in mean age, body mass index, duration of infertility, type of infertility, number of embryos transferred and endometrial thickness on the day of beginning progesterone therapy. 

**Table 1:** Baseline characteristics and clinical data at the two study groups

**Variation**	**GnRH agonist** **(n = 60)**	**No GnRH agonist** **(n = 40)**	**P value**
Total numbers of cycles	60	40	
Total numbers of ET	60	38	
Cancelled cycles	0	2	
Age, years	30 ± 5.1	32 ± 5.9	0.065
Duration of infertility, years	8.9 ± 5.1	9.8 ± 5.6	0.429
Body mass index, kg/m^2^	26.5 ± 4.3	27.3 ± 3.3	0.308
Type of infertility			
Primary	45 (76.3)	31 (79.0)	0.712
Secondary	14 (24.0)	8 (21.0)	0.724
Endometrial thickness (mm)	10 ± 1.7	10.7 ± 2.9	0.229
Number of embryos transferred	136	98	0.516
Number of embryos transferred/ET	2.2	2.5	0.805

ET = embryo transfer

Results are presented as mean ± SD or number (%)

Two cycles were cancelled in B Group because the endometrium failed to reach a thickness of at least 7 mm, whereas no cycle was cancelled in group A. The average number of embryos transferred per patient was 2.2 in the group received GnRH agonist compared to 2.5 in the group did not receive the agonist with no difference (p = 0.805).

Comparing outcome of FTET between the two groups scheduled for receiving GnRH agonist (Table 2) showed no significant difference in the rate of implantation (6.7% versus 10.0%), the rate of chemical pregnancy (21.7% versus 22.5%), clinical pregnancy rate (15.0% versus 17.5%), and also ongoing pregnancy (13.3% versus 12.5%).

**Table 2:** Clinical outcomes after frozen-thawed embryos were transferred

**Characteristic ** **Value**	**GnRH ** **agonist** **(n = 60)**	**No GnRH ** **agonist** **(n = 40)**	**P ** **value**
Implantation rate	4 (6.7%)	4 (10.0%)	0.862
Chemical pregnancies/ET	13 (21.7%)	9 (22.5%)	0.885
Clinical pregnancies/ET	9 (15.0%)	7 (17.5%)	0.588
Ongoing pregnancies /ET	8 (13.3%)	5 (12.5%)	0.162

## Discussion

Optimization of pregnancy rate direct depends to synchronization of developing endometrium and embryo. In addition to normal growth of embryo, preparation of endometrium is necessary that can be simplistically obtained by natural cycle FTET through production ofendogenous sex steroid from active follicles.

This pathway can be activated by administrating exogenous sexual hormones such as GnRH agonists. In fact, the use of these agonists may increase endometrial receptivity (12, 13). In a form of FTET as artificial cycle FTET, estrogen and progesterone are co-administered sequentially to imitate the endocrine exposure of the endometrium. Initially, estradiol is used for proliferation of endometrium till achieving a 7 to 9 mm endometrial thickness in ultrasonography assessment (14). However, the use of both sexual hormones may not guarantee pituitary suppression sufficiently. In this regard, co-treatment with GnRH agonist is now considered to down-regulate the activity of pituitary as well as to inhibit follicular growth. However it remains uncertain whether exogenous steroids with and without pretreatment by the use of GnRH agonist is superior to another regarding pregnancy rate.

According to our interventional study, there was no difference in both clinical and chemical pregnancy rates between the two pointed protocols. In some previous studies, the rate of pregnancy was indicated not to differ regarding the use of a GnRH agonist (15). In a study by Azimi Nekoo et al, a depot GnRH agonist was administered in the mid-luteal phase of previous cycle in a group and steroid supplementation without prior pituitary suppression in another group indicating similar chemical and clinical pregnancy rates between the two groups (15). In another study by Dal Prato et al, the two groups receiving exogenous steroids with and without pretreatment by GnRH agonist, no difference was revealed in the number of embryos transferred per patient, rates of pregnancy, the rate of abortion, and also the rate of implantation (16). In total, as shown in our survey, the administration of GnRH agonists may not have effective role on quality of embryo and endometrial development indicated by the rate of pregnancy. In other word, although administrating GnRH agonists is very beneficial to prevent spontaneous ovulation and cycle cancelation, some studies could not demonstrate advantages of adding GnRH agonists to standard protocol to improve reproductive outcome and implantation rate (17, 18). Even, it has been revealed that the root of administrating GnRH agonists cannot affect the pregnancy outcome after embryo transfers (19). Therefore it is suggested that suppression with a GnRH agonist is not necessary for endometrial preparation for FTET. Skipping administration of a GnRH agonist makes the procedure simpler and cheaper. 

Endometrial preparation is quicker, and the patient or medical staff can still choose the date of transfer. When the pituitary is not suppressed by using a GnRH agonist, it is very important to start estradiol treatment in the early follicular phase on day 1 or day 2. With this approach, although initial follicular activity is sometimes present, spontaneous ovulation seems to be inhibited. Starting estradiol treatment after the third day of the cycle might lead to an increased incidence of LH surge and luteinization of the endometrium (9). Step-up protocol using 100 g to 300 or 400 g of estradiol administered by transdermal patches has been adequate for inhibition of ovulation and endometrial preparation (20). We believe that more physiologic estrogenic stimulation of endometrium that mimics the hormonal pattern of a spontaneous cycle is more suitable for endometrial development. Minimum thickness of a receptive endometrium should be 5-8 mm (21).

In summary; this study presents a flexible, simple, and inexpensive protocol for controlled preparation of the endometrium in women with functioning ovaries undergoing frozen-thawed ET. Using this protocol for the transfer of thawed embryos has a similar success rate. Its application for an egg-donation program, however, has yet to be evaluated.

## Conclusion

Endometrial preparation for FTET using GnRH agonists appears to be as effective as FTET without administrating these agonists.
